# Seasonal Response of Grasslands to Climate Change on the Tibetan Plateau

**DOI:** 10.1371/journal.pone.0049230

**Published:** 2012-11-16

**Authors:** Haiying Yu, Jianchu Xu, Erick Okuto, Eike Luedeling

**Affiliations:** 1 Key Laboratory of Biodiversity and Biogeography, Kunming Institute of Botany, Chinese Academy of Sciences, Kunming, China; 2 World Agroforestry Centre, East-Asia Program, Kunming, China; 3 World Agroforestry Centre, Gigiri, Nairobi, Kenya; Duke University, United States of America

## Abstract

**Background:**

Monitoring vegetation dynamics and their responses to climate change has been the subject of considerable research. This paper aims to detect change trends in grassland activity on the Tibetan Plateau between 1982 and 2006 and relate these to changes in climate.

**Methodology/Principal Findings:**

Grassland activity was analyzed by evaluating remotely sensed Normalized Difference Vegetation Index (NDVI) data collected at 15-day intervals between 1982 and 2006. The timings of vegetation stages (start of green-up, beginning of the growing season, plant maturity, start of senescence and end of the growing season) were assessed using the NDVI ratio method. Mean NDVI values were determined for major vegetation stages (green-up, fast growth, maturity and senescence). All vegetation variables were linked with datasets of monthly temperature and precipitation, and correlations between variables were established using Partial Least Squares regression. Most parts of the Tibetan Plateau showed significantly increasing temperatures, as well as clear advances in late season phenological stages by several weeks. Rainfall trends and significant long-term changes in early season phenology occurred on small parts of the plateau. Vegetation activity increased significantly for all vegetation stages. Most of these changes were related to increasing temperatures during the growing season and in some cases during the previous winter. Precipitation effects appeared less pronounced. Warming thus appears to have shortened the growing season, while increasing vegetation activity.

**Conclusions/Significance:**

Shortening of the growing season despite a longer thermally favorable period implies that vegetation on the Tibetan Plateau is unable to exploit additional thermal resources availed by climate change. Ecosystem composition may no longer be well attuned to the local temperature regime, which has changed rapidly over the past three decades. This apparent lag of the vegetation assemblage behind changes in climate should be taken into account when projecting the impacts of climate change on ecosystem processes.

## Introduction

Vegetation has an essential function in regulating carbon and energy exchange through phenology [1]–[4], photosynthetic activity [5], [6], autotrophic and heterotrophic respiration [6]and other processes. Several studies have considered changes in vegetation productivity as the primary cause of variation in terrestrial net carbon uptake [6]–[9]. Recent climate change has exerted significant influences on terrestrial ecosystems and impacts are projected to be even greater in the future [10]. Considerable evidence shows that temperature in the late 20^th^ century was anomalous in relation to the last 1,800 years [11], and the period from 2000 to 2009 was the warmest since instrumental measurements began [6]. Vegetation growth in mid to high latitudes of the Northern Hemisphere is very sensitive to temperature changes [12], [13], and a climate-driven greening trend has been well-documented since the 1980s [14]–[16]. With its Northern Hemisphere mid-latitude position and high elevation, the Tibetan Plateau is experiencing even faster temperature increases than the global average [17]with net primary productivity (NPP) increasing remarkably, especially in alpine meadows [18].

Assessments of the impact of climate change on plant growth need to examine changes in mean growth rates, seasonality and variability [19]. The instrumental climate record shows high variation in seasonal temperature change: winter months have warmed more rapidly than summer months; night-time temperatures are more affected than temperatures during the day [19], [20]; and the number of frost days (minimum air temperature<0°C) has declined [21]. Overall change in precipitation is harder to generalize, because of substantial temporal and spatial variation [20]. In ecosystems that receive less than 600 mm mean annual precipitation, primary production is largely constrained by water availability, which is, in part, controlled by the nature and timing of rainfall events [22]–[25].

**Figure 1:**
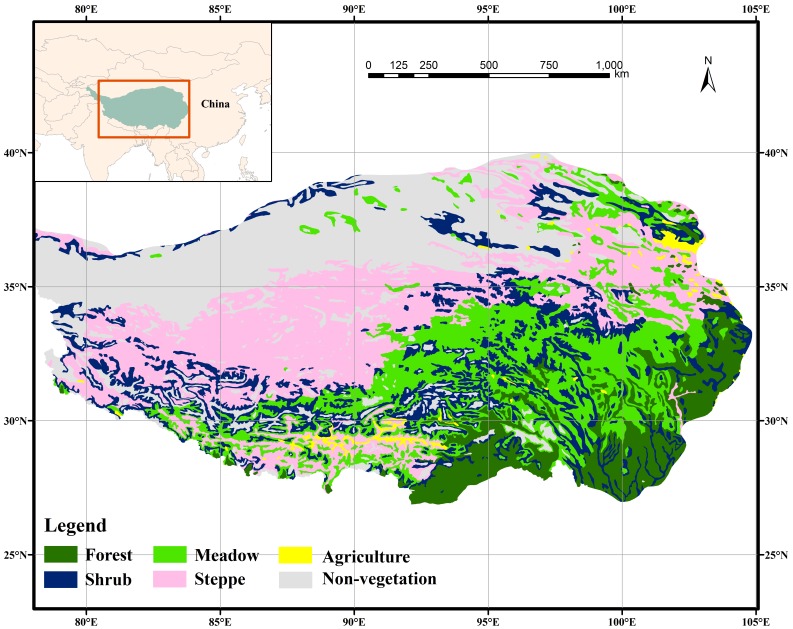
Overview map of the Tibetan Plateau in southwestern China, showing the different vegetation types present in the region.

Different ecosystems have been shown to respond differently to climate change, and the mechanisms underlying ecological responses to climate, and even the direction of these responses themselves, are subject to ongoing debate. While many sites among the more populated areas of the world have long records of plant phenology and productivity that can be mined for insights into climate responses, no such records exist for the Tibetan Plateau. Yet this region is not only of crucial importance for the world’s climate system, it has also experienced strong climate changes in recent years, most notably a pronounced warming trend [26], [27]. For the Tibetan Plateau, spring phenology advanced before the mid-1990s and retreated afterwards, resulting in a shortening of the growing season [28]. This trend resulted from the combined effects of temperature changes in different seasons [28]. The same climatic drivers are likely to not only affect phenology but also photosynthetic capacity and vegetation activity, yet no research published to date has investigated these effects. The aim of this study is thus to investigate the interannual change trend of grassland activity and to clarify how seasonal grassland growth has responded to climate change from 1982 to 2006.

## Methods

### Study Area

The Tibetan Plateau is located in southwestern China, where it covers an area of approximately 257.24×10^4^ km^2^ [29]. Elevation averages more than 4000 m above mean sea level. The regional climate is influenced by the Indian Ocean monsoon in summer and by westerly circulation in winter. Meadow and steppe are the two major vegetation types, occupying 24% and 27% of the total area of the Plateau, respectively (Fig. 1).

**Figure 2:**
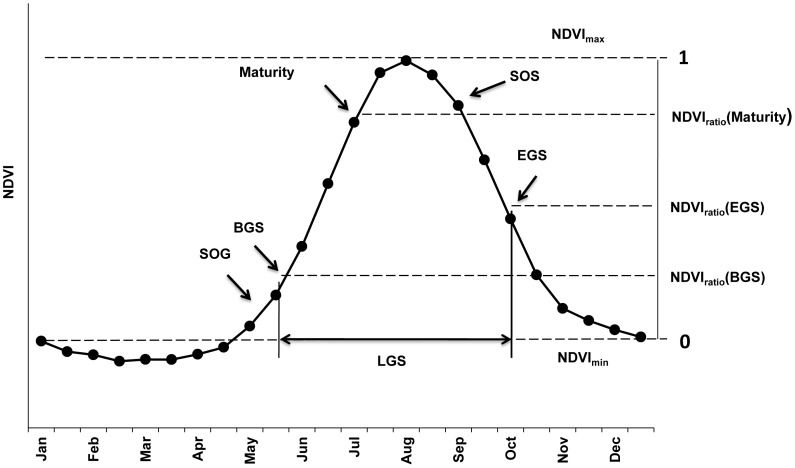
Illustration of the NDVI ratio method for determining the timing of vegetation stages. All NDVI values are expressed relative to the range between minimum (substituted here by the mean of all positive NDVI values during February and March) and maximum NDVI of the season. Rules for determining the timing and beginning of the Start of Green-up (SOG), the Beginning of the Growing Season (BGS), Maturity, Start of Senescence (SOS) and the End of the Growing Season (EGS) are given in the text.

### Vegetation Analysis

The Normalized Difference Vegetation Index (NDVI) is the most widely used satellite-derived metric for vegetation monitoring and ecological modeling, providing a good measure of vegetation growth, or photosynthetic capacity. In this study, we used the dataset created by the Global Inventory Modeling and Mapping Studies group (GIMMS) [30], [31], which was downloaded from the Global Land Cover Facility of the University of Maryland (www.landcover.org). This dataset comprises 15-day composites of NDVI data derived from imagery obtained by NOAA/AVHRR. The GIMMS data has a spatial resolution of 8 km and provides continuous coverage between 1982 and 2006.

**Figure 3:**
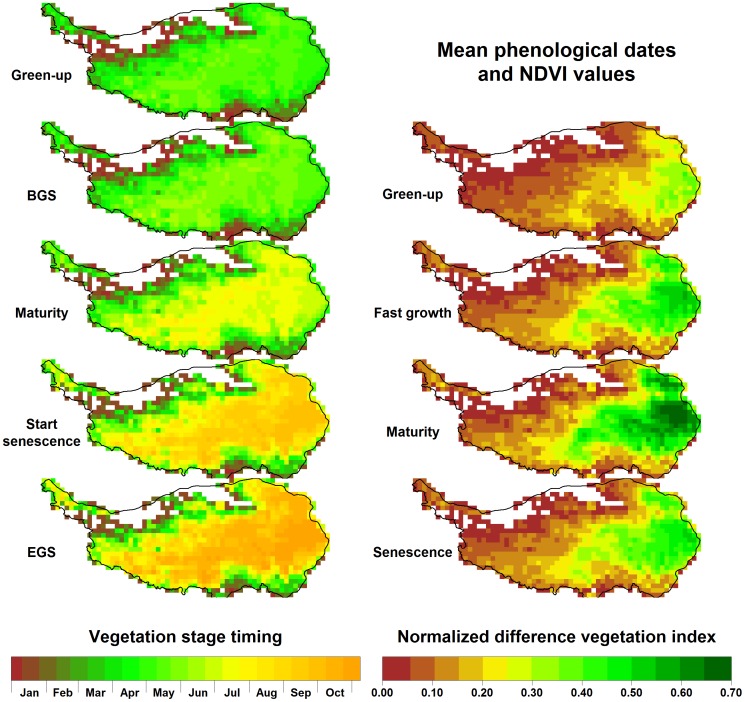
Mean phenological dates and vegetation activity during different growth stages on the Tibetan Plateau, based on NDVI data between 1982 and 2006.

For each pixel of the GIMMS dataset that was located on the Tibetan Plateau, all 600 NDVI values over the 25 year time span covered by the images (24 images per year) were extracted and used for detection of phenological phases. Grassland phenology was evaluated using the NDVI ratio method [32]. In this procedure, the minimum and maximum NDVI for each year are determined and all vegetation activity measurements are expressed relative to the range spanned by these two values (Fig. 2). To avoid the potentially perturbing influence of snow cover, minimum NDVI was substituted by the mean of all NDVI records during February and March that were greater than zero. This procedure resulted in vegetation dynamics curves for each pixel of the dataset, with a minimum NDVI ratio value of 0 in February or March and a maximum of 1 at the time of greatest photosynthetic capacity. From these curves, phenological dates were determined by the following rules:

- The beginning of the growing season (BGS) was defined as the date when the NDVI ratio first exceeded 0.2 and was followed by three consecutive dates of increasing NDVI ratio [28].
- The start of vegetation green-up, which occurs before greening can be detected by remote sensing, was set to the date, at which the GIMMS-NDVI image before the BGS date was captured. The green-up phase was thus assumed to last for approximately 30 days and comprise two GIMMS-NDVI images.
- The date of vegetation maturity was defined as the date when the NDVI ratio first exceeded 0.8.
- The end of the growing season (EGS) was interpreted as the first date when the NDVI ratio dropped below 0.6, when this was followed by three consecutive dates of decreasing NDVI ratio [28].
- The start of grassland senescence was set to the date, at which the penultimate GIMMS-NDVI image before the end of the growing season was captured. This resulted in a senescence phase lasting for approximately 45 days and comprising 3 GIMMS-NDVI images.

**Figure 4:**
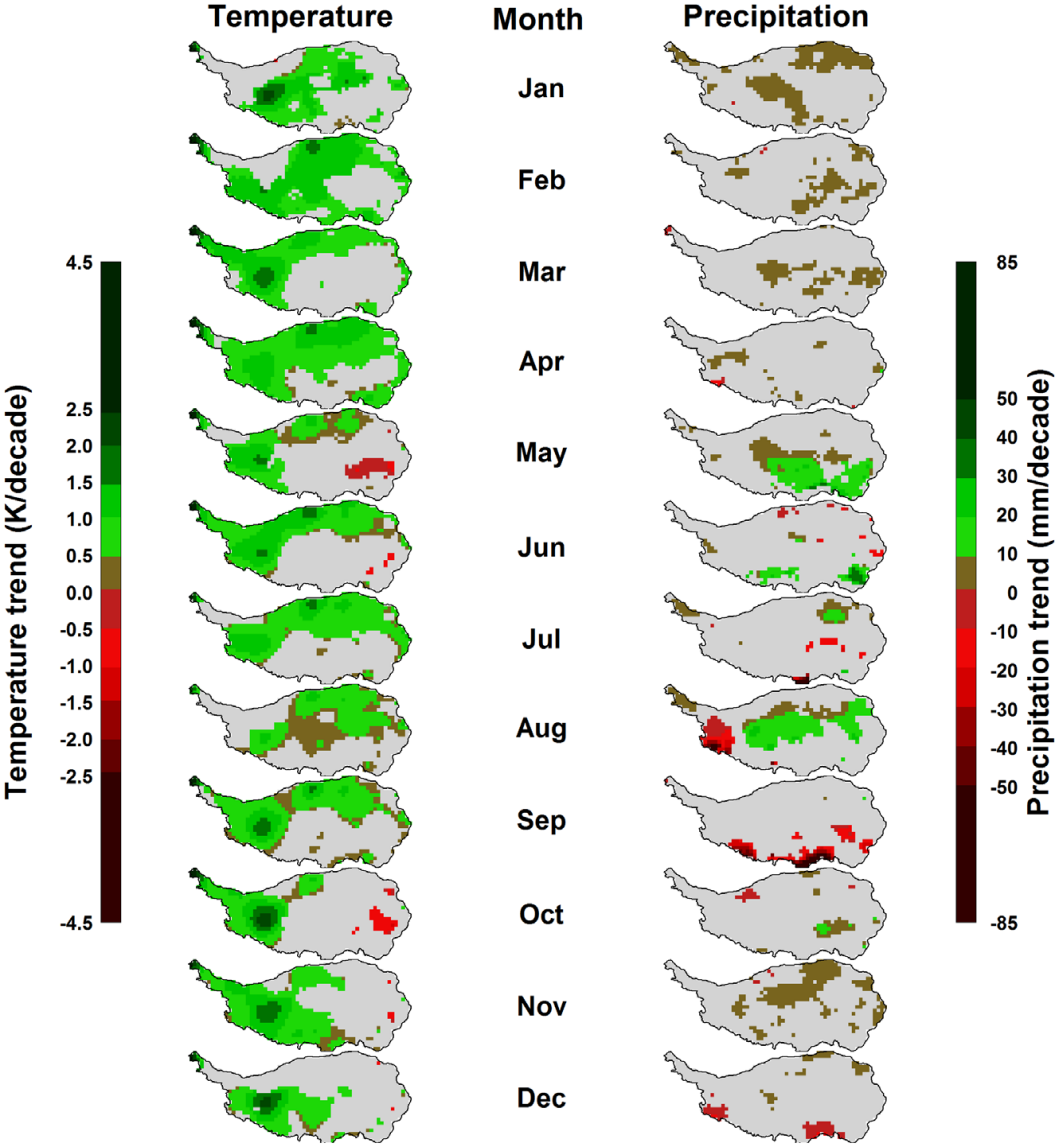
Trends in monthly mean temperature (left) and precipitation (right) on the Tibetan Plateau between 1982 and 2006. Gray areas indicate regions, for which no significant trends were detected by the Mann-Kendall test at p<0.1.

**Figure 5:**
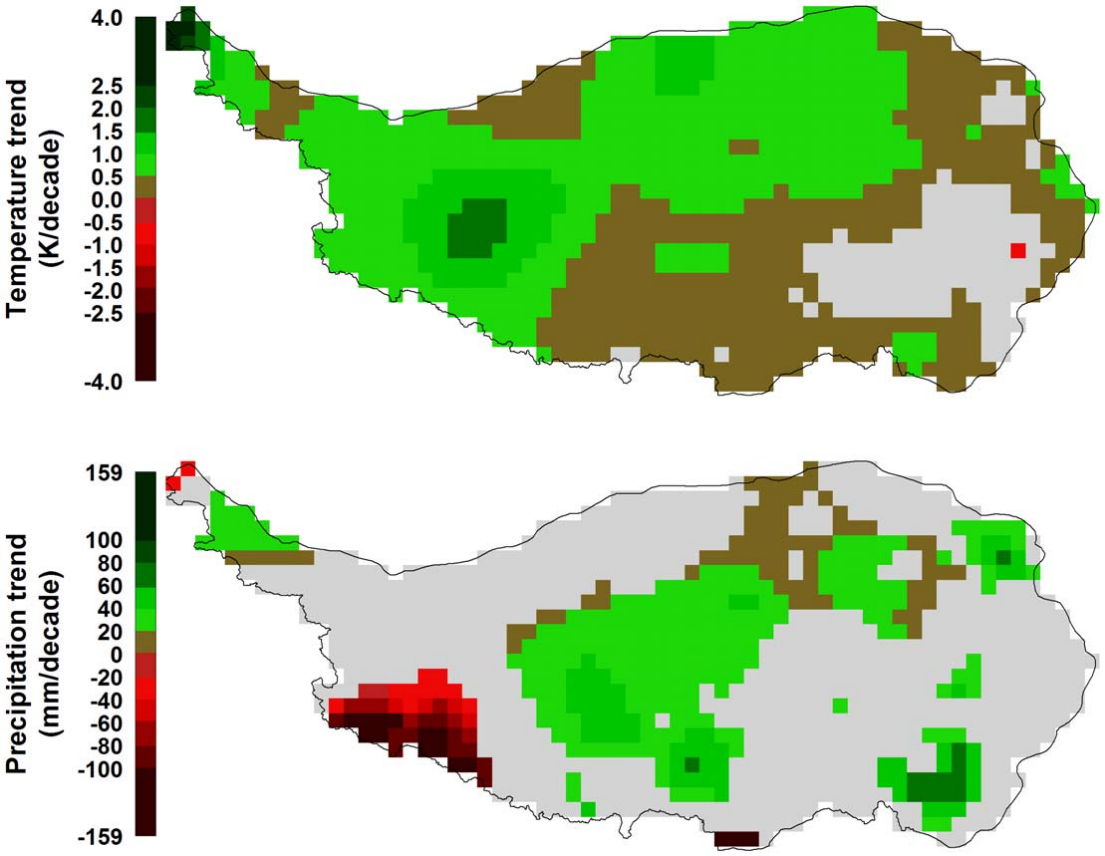
Trends in annual mean temperature (top) and total annual precipitation (bottom) on the Tibetan Plateau between 1982 and 2006. Gray areas indicate regions, for which no significant trends were detected by the Mann-Kendall test at p<0.1.

The accuracy of procedures used to determine BGS and EGS has been assessed by Yu et al. [28], based on observations from 22 grassland monitoring stations in Qinghai Province. The Root Mean Squared Error (RMSE) of BGS dates was 10.6 days, while RMSE for EGS dates was slightly higher at 13.5 days. This level of accuracy was deemed sufficient for reliable detection of phenological events throughout the Tibetan Plateau.

Not in all cases were vegetation dynamics derived with these procedures coherent, so all pixel/year combinations where phenological dates did not occur in the correct sequence (start of green-up, BGS, maturity, start of senescence, EGS) were excluded from further analysis. Furthermore, all pixel/year combinations, for which mean NDVI between the beginning and end of the growing season was less than 0.1, were excluded from further processing. Such values indicate very sparse vegetation, which is likely to produce erroneous results. Mean NDVI values were then calculated for the following phenological phases: Green-up (start of green-up until the BGS date), fast growth (two weeks after BGS until two weeks before maturity), mature (maturity until two weeks before the start of senescence), and senescence (start of senescence until EGS).All vegetation analyses resulted in annual grids for the timing of each vegetation phase, as well as the mean NDVI for each vegetation stage, with a spatial resolution of 8 km.

**Figure 6:**
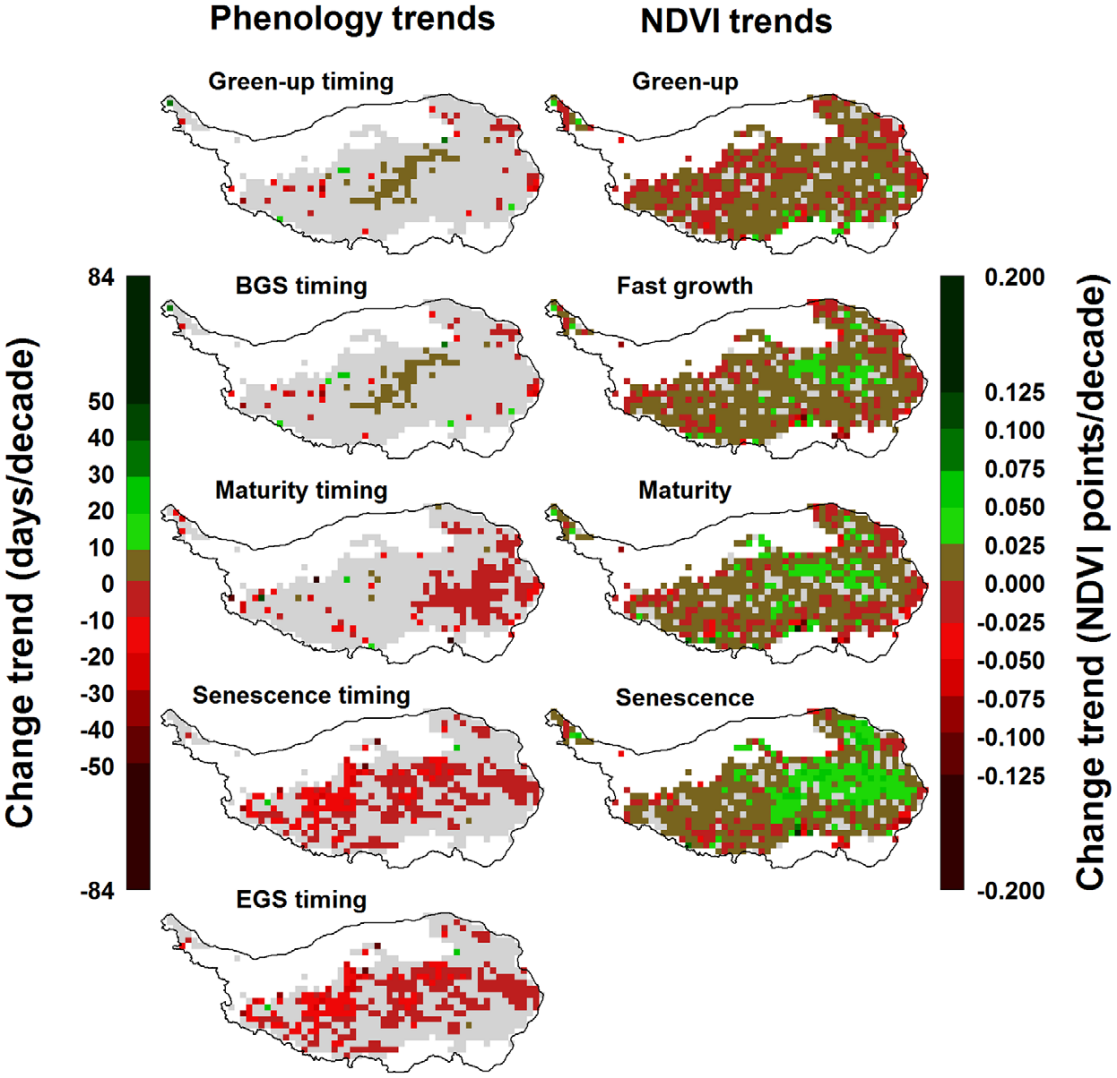
Trends in phenological dates (left) and NDVI (right) on the Tibetan Plateau between 1982 and 2006. Gray areas indicate regions, for which no significant trends were detected by the Mann-Kendall test at p<0.1.

### Climate Data

Global gridded datasets of mean monthly temperature and precipitation were obtained from the University of Delaware’s 1900–2008 Gridded Monthly Time Series database. This dataset covers the entire terrestrial area of the planet at a spatial resolution of 0.5° [33], [34]. Since the climate datasets were coarser than the vegetation data, the latter dataset had to be resampled to the resolution of the climate data, so that meaningful correlation analysis became possible [35]. All vegetation layers were first aggregated into 5×5 pixel means and then resampled to the resolution of the climate data, using bilinear interpolation. The resulting dataset covered the Tibetan Plateau with 1021 pixels. Following the resampling, vegetation data were combined with temperature and precipitation records for the year of the vegetation observations, as well as the preceding year.

**Figure 7:**
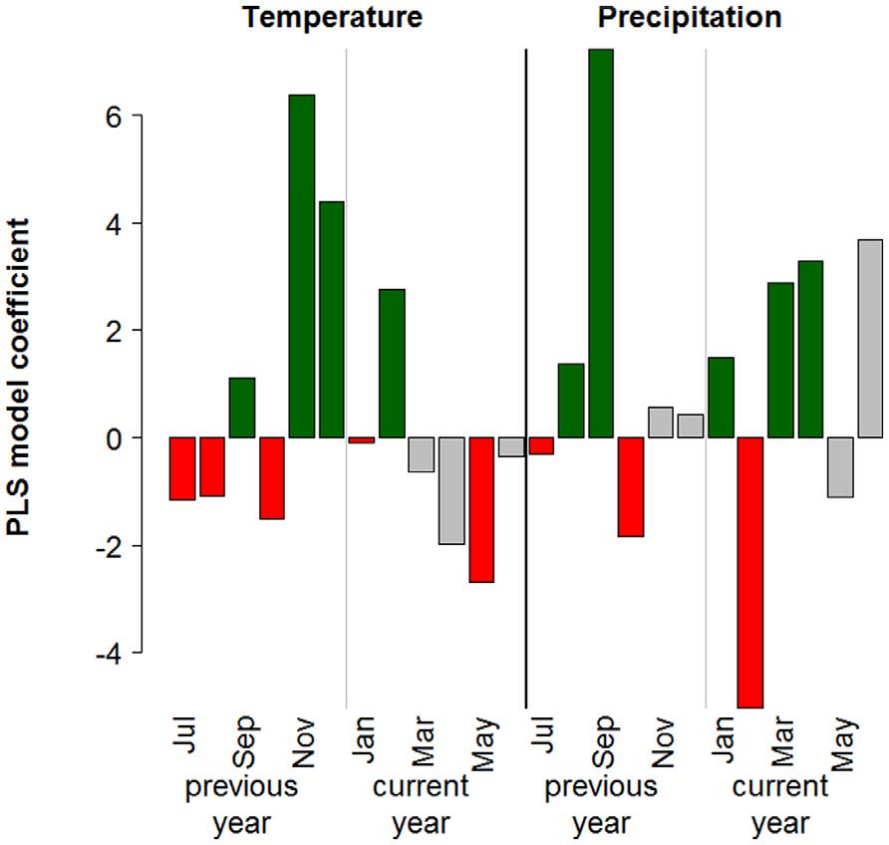
Results from Partial Least Squares regression analysis for one pixel of the analysis grid, showing results for the beginning of the growing season (BGS). Green bars indicate variables, for which a positive correlation with late BGS dates was determined (possibly indicating a delaying effect of high temperatures or precipitation during this month). Red bars indicate a negative correlation (indicating an advancing effect of high temperatures or rainfall). Gray bars are drawn for variables, for which the variable-importance-in-the-projection score was<0.8, indicating that the variable is not important. This color scheme is used for all subsequent maps.

### Trend Analysis

All climate and vegetation data were subjected to trend analysis. For detecting the presence of significant trends, we applied the non-parametric Mann-Kendall test [36], which tests the null hypothesis that no trend is present in the data. This test does not require a particular distribution of data points or residuals and should thus produce valid results for all pixels of the study region. Where significant trends were detected, a linear model was fitted to the data, and the slope of the resulting regression equation was used to illustrate the rate of change over the study period. For the Mann-Kendall test, we rejected the null hypothesis of no trend in the time series at an error probability level of 0.1.

### Identification of Climatic Drivers

Partial Least Squares (also known as Projection to Latent Structures; PLS) regression was applied for screening for statistical relationships between independent climate variables and dependent vegetation variables. This method first determines latent factors, a variant of principal components, to reduce the dimensionality of the independent (and optionally also the dependent) variables [37]. It then uses these factors as independent factors in a linear model to explain variation in the dependent variables. This method is frequently used in remote sensing [38]–[40], and has recently been shown to have potential for analysis of high-resolution climate data [41]–[43].

**Figure 8:**
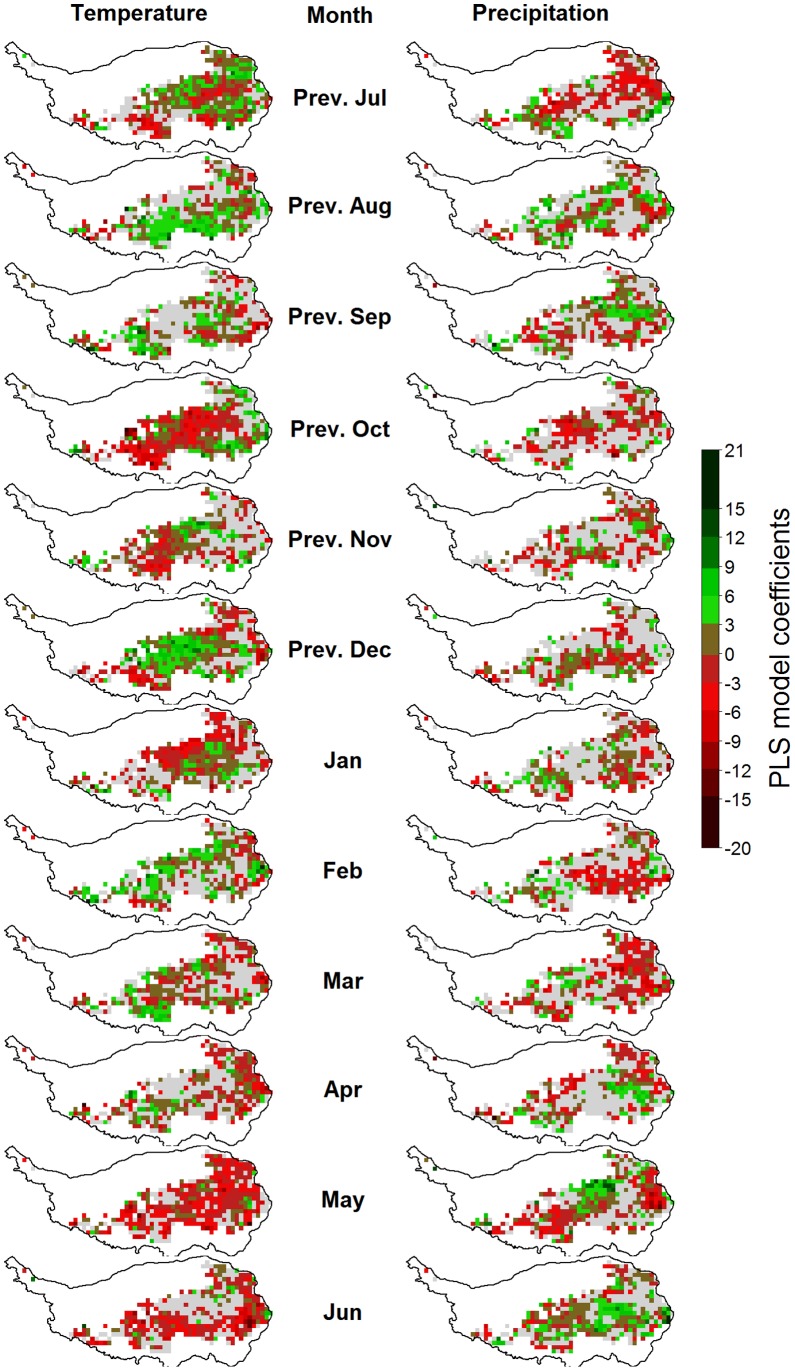
Correlations of monthly temperatures (left) and precipitation (right) with the beginning of the growing season (BGS) on the Tibetan Plateau, according to Partial Least Squares (PLS) regression. For each variable, pixels for which the variable-importance-in-the-projection score was<0.8 are shown in gray. Pixels with insufficient data for PLS analysis are shown in white.

**Figure 9:**
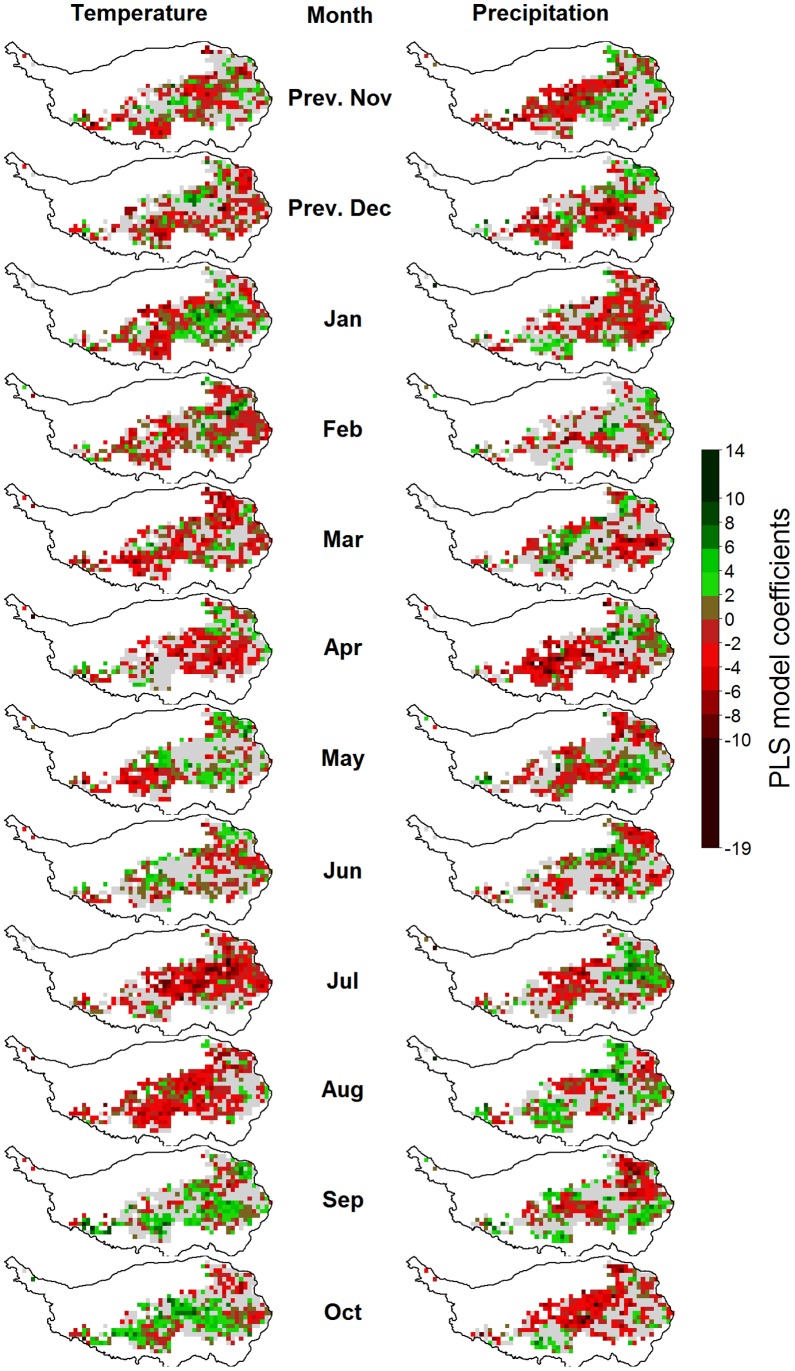
Correlations of monthly temperatures (left) and precipitation (right) with the timing of grassland senescence on the Tibetan Plateau, according to Partial Least Squares (PLS) regression. For each variable, pixels for which the variable-importance-in-the-projection score was<0.8 are shown in gray. Pixels with insufficient data for PLS analysis are shown in white.

All available vegetation variables were related in separate PLS analyses to climate variables. The dependent variable in a given PLS model was thus one of the vegetation variables (e.g. beginning of the growing season or NDVI during senescence), while independent variables were temperature and precipitation in the twelve months preceding the phenological event in question. For vegetation activity assessments of growth stages, weather conditions during the 12 months preceding the end of the respective growth stage were used as independent variables. Because the timing of vegetation events varied throughout the Tibetan Plateau, ‘typical’ phenological dates were used for deciding which months to include in the set of independent variables. These typical dates were determined by the 75^th^ percentile of the distribution of each set of phenological dates. In other words, the typical date for a phenological event was assumed to be the date, at which this event had occurred in 75% of pixel/year combinations, for which valid vegetation development curves could be constructed. Where these dates fell before the 15^th^ of a given month, this month was not included in the 12-month time window, meaning that the last month included in the set of independent variables was the month preceding the typical phenological date. Where typical dates were after the 15^th^ of the month, the respective month was the last month included in the set of independent variables.

The number of latent factors to be included in PLS models was determined by cross-validation, as the minimum number of factors that explained at least 90% of the variation in the dependent variable. Where 10 latent factors were insufficient for reaching this threshold, the analysis was run with 10 factors.

Separate PLS regression analyses were carried out for each pixel of the dataset, after centering and scaling all data for the respective pixel. The pixel-scale approach was preferred to combined analysis of whole biomes [28], because variation of ecosystem composition within a biome, especially along climatic gradients, might compromise the usefulness of the PLS analysis. PLS regression evaluates the effects of departures from ‘normal’ conditions among the independent variables. If vegetation data from large, climatically heterogeneous areas were combined into one analysis, there would not be clearly defined ‘normal’ temperature and precipitation conditions, so that interpreting results from PLS regression would be difficult. Typical outputs from the PLS analysis are a Variable-Importance-in-the-Projection (VIP) statistic and model coefficients for the PLS regression model, for each independent variable. In the present analysis, a VIP value and a model coefficient are calculated for temperature and precipitation for each of the preceding twelve months, for each of the vegetation parameters. According to Wold [37], variables with VIP scores of 0.8 or greater are considered important for the model. In presenting results from the analysis, we only show model coefficients for variables that were important according to this criterion. Model coefficients indicate the direction and strength of the effects of high values for a given independent variable on the dependent variable. For example, a high and positive model coefficient for temperatures in January, combined with a high VIP score, indicates that high temperatures during this month show a positive correlation with the dependent variable, implying in this case a delaying effect on vegetation timing or a positive effect on the NDVI. Negative model coefficients indicate that high values for the respective variable are related with an advance in phenology or with lower vegetation activity.

Pixel-scale results were mapped to show the spatial variation in the strength and direction of climatic drivers on the various vegetation variables. All procedures were implemented in R programming language [44], drawing heavily from the ‘raster’ package [45] and the ‘pls’ package [46].

## Results

### Vegetation Dynamics on the Tibetan Plateau

Based on the means of phenological dates for the green-up phase over 25 years, greening of the Tibetan Plateau typically occurs between April and May in most places. It is followed by the beginning of the growing season in May or June (Fig.3). In most parts of the plateau, plants reached their mature stage in July, and began to senesce in September. The growing season ended in October in most grassland regions of the Tibetan Plateau (Fig. 3). The cooler steppe regions lagged substantially behind the more vigorous meadow vegetation of the eastern part of the plateau. NDVI values were naturally low during green-up at between 0.00 and 0.35 (Fig. 3). In the meadow environment, NDVI peaked during maturity at up to 0.7, while few steppe locations exceeded NDVI values of 0.45. During senescence, NDVI dropped substantially to about half the level of peak NDVI throughout the plateau. The analyses showed a pronounced east-west gradient in mean NDVI during all vegetation phases, with the cooler and dryer steppe environment of the Western Tibetan Plateau showing low vegetation intensity. For most of the northwestern region, mean NDVI during the growing season was less than 0.1, leading to exclusion of these areas from further analysis (Fig. 3).

### Trends in Vegetation and Climate

Temperature showed increasing trends throughout much of the Tibetan Plateau, for most months of the year (Fig. 4).In particular in the northern and western regions, these trends were significant according to the Mann-Kendall test (at p<0.1). In the areas of densest vegetation, trends were mostly not significant, and in October and May, small regions in the southeast of the plateau even showed a significantly decreasing trend. Where significant increasing trends were present, these mostly ranged around+1K per decade (Fig. 4). For precipitation, which exhibits stronger interannual variation than temperature, few significant trends were detected (Fig. 4). Somewhat consistent patterns across larger areas were slight precipitation increases in January, May and August.In some regions of the southern plateau, precipitation decreased in August and September. Trends in mean annual temperature and total annual precipitation were significant throughout most of the Tibetan Plateau (Fig. 5). Most of the study region, with the exception of the south-eastern corner, showed significant temperature increases by between 0 and+1.5K per decade. Slightly increasing annual precipitation totals of up to+60 mm per decade were found throughout approximately half of the study region, while a small region in the south-west of the plateau exhibited significant precipitation losses over time (Fig. 5).

For the timing of early-season phenological stages, significant trends were detected for few pixels of the analysis grids. Green-up and the beginning of the growing season showed slight delays in some parts of the central plateau over the 25-year period that was analyzed, but these were rarely significant (Fig. 6). Grassland maturity, senescence and the end of the growing season predominantly showed advancing trends, which in many places were significant. For 16% of the plateau area, advances in senescence and end of the season occurred at a rate of more than one week per decade, and for 4% of the study region these stages advanced by more than two weeks per decade (Fig. 6). For NDVI values for all growth stages, most pixels of the analysis grid showed significant increases over time, mostly ranging around+0.05 NDVI points per decade for the green-up, fast growth, maturity and senescence phases (Fig. 6).

### Climatic Drivers of Phenology

Partial Least Squares regressions were carried out for all 1021 pixels of the analysis grid. Figure 7shows the typical output of such an analysis. As noted in the methods description, the PLS procedure produces two relevant metrics for each independent variable: the VIP score, which indicates the importance of a variable in the PLS model, and the model coefficient, which quantifies the effect. In the diagram, bars corresponding to independent variables whose VIP score is below the threshold of 0.8 are shown in grey. The remaining bars are colored in red for negative model coefficients and in green for positive ones (Fig. 7).

**Figure 10:**
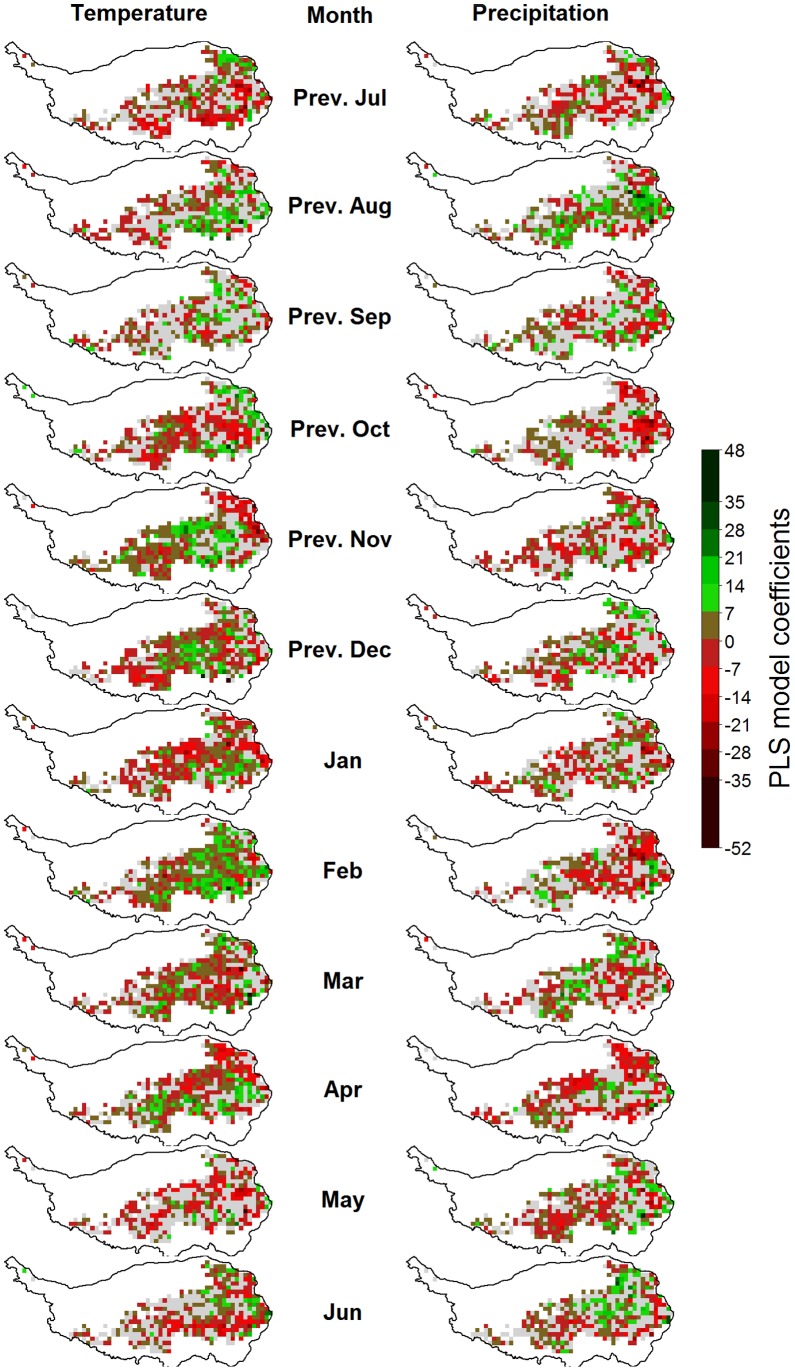
Correlations of monthly temperatures (left) and precipitation (right) with vegetation activity during the green-up phase (as measured by the NDVI) on the Tibetan Plateau, according to Partial Least Squares (PLS) regression. For each variable, pixels for which the variable-importance-in-the-projection score was<0.8 are shown in gray. Pixels with insufficient data for PLS analysis are shown in white.

**Figure 11:**
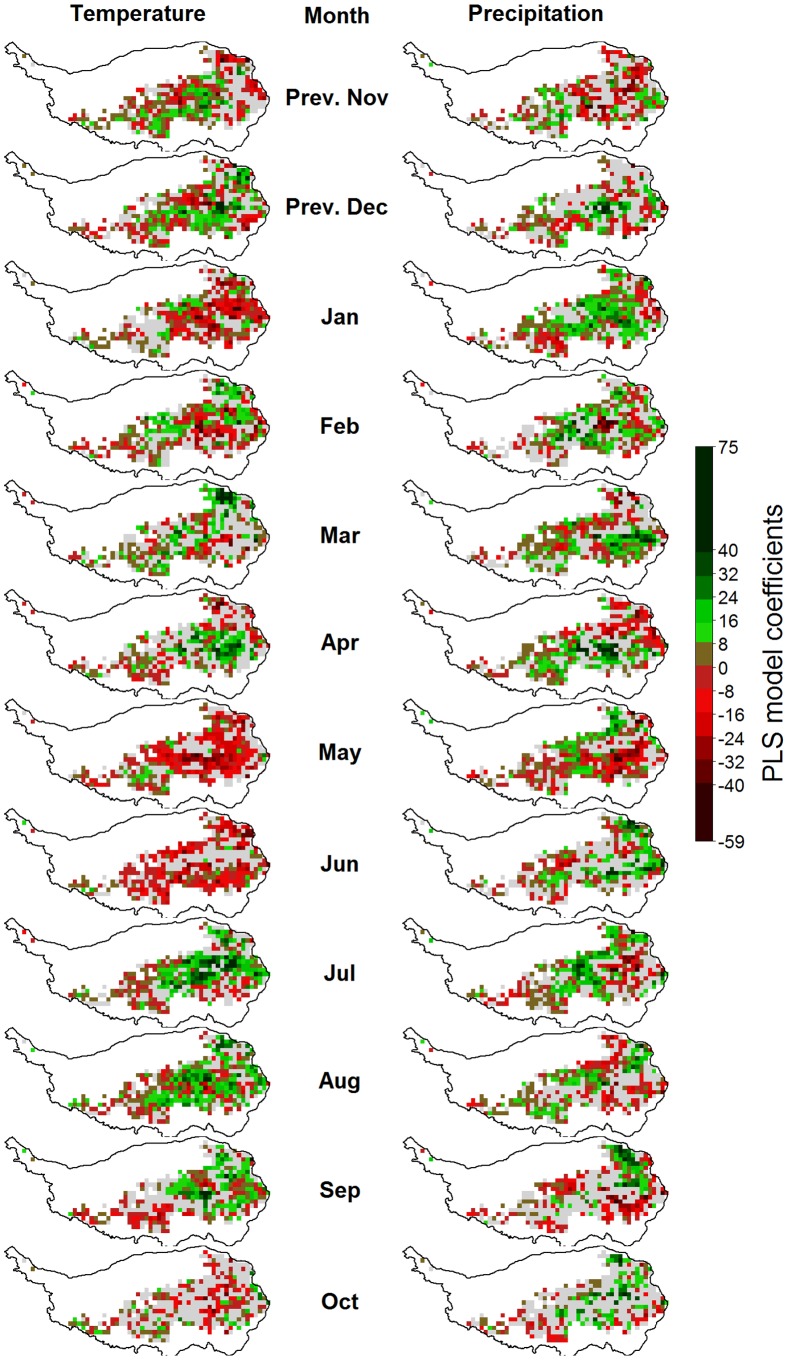
Correlations of monthly temperatures (left) and precipitation (right) with vegetation activity during grassland senescence (as measured by the NDVI) on the Tibetan Plateau, according to Partial Least Squares (PLS) regression. For each variable, pixels for which the variable-importance-in-the-projection score was<0.8 are shown in gray. Pixels with insufficient data for PLS analysis are shown in white.

Such outputs were produced for each pixel and mapped according to the color code used in the example figure (Fig. 7). In Figures 8, 9, 10, 11 and Figures S1, S2, S3 in the appendix, shades of green and red indicate the strength of the effects. As in the example, grey pixels in the maps signify that data was available for a given pixel, but the respective independent variable was not important according to the VIP statistic. Where pixels are white, insufficient vegetation data was available for running the PLS procedure.

### Drivers of the Timing of Phenological Stages

For the beginning of the growing season, PLS regression indicated strong but variable influences of temperatures in different months (Fig. 8). Warm conditions in the previous July, August and December, as well as in the January, February and March preceding the beginning of the growing season were correlated with delayed BGS dates for many pixels. Warm conditions in May and June appeared to be the main advancing drivers of BGS dates. During the other months, effects were mixed. Relationships between precipitation and BGS dates were inconsistent for all months.

Early plant maturity was related with warm conditions in the previous October, as well as in January, April, May, June, July and August (see the legend for Figure S1). A slight delaying effect of warm conditions in the previous December, as well as in February, was indicated by positive model coefficients for temperatures during these months. Precipitation effects varied, but more often than not high precipitation appeared to advance plant maturity. Only high rainfall in July was clearly related to delays in plant maturity.

An early end of the growing season was primarily related with high temperatures in July and August, while a late cessation of growth was linked with warm conditions in September and October (Fig. 9). Before May, high temperatures appeared to generally have an advancing effect, but this was not consistent. Precipitation effects were mixed and cannot easily be generalized. Since the beginning of the green-up and senescence stages were derived from the EGS and BGS dates by simply subtracting four and two weeks, respectively, PLS results for BGS and EGS also indicate climatic drivers of the derived phenological stages.

### Drivers of Vegetation Activity

Vegetation activity during the green-up phase, as expressed by the NDVI, was positively correlated with high temperatures during the previous November and December, as well as January, February and March (Fig. 10). Effects of temperatures during other months, as well as precipitation during all months, did not show spatially coherent patterns.

For the stage of fast growth, between the beginning of the growing season and plant maturity, high temperatures in November, December, January, February, July and August showed a positive effect, while warm conditions in March, May and possibly June were related with low NDVI values (see the legend for Figure S2). High rainfall appeared to have a positive effect on vegetation intensity during fast growth when it occurred in May or June. In contrast, high rainfall in July appeared to reduce photosynthetic capacity.

NDVI in the mature stage was mainly related to temperatures during May and June, when high temperatures were linked with low vegetation activity at plant maturity (see the legend for Figure S3). Warm conditions during July and August, in contrast, were related with high NDVI values. Wet conditions in May, June and July were positively related with high NDVI during the mature season in some locations.

Vegetation activity during plant senescence was related with high temperatures in May and June, which appeared to reduce NDVI values (Fig. 11). Warm conditions in July, August and September seemed to have a promoting effect on vegetation. Precipitation effects were again inconsistent, but generally, high precipitation during most months was related to late cessation of growth.

## Discussion

### Climatic Trends

Most parts of the Tibetan Plateau have experienced significant temperature increases over the past 25 years (83% of all pixels, according to the Mann-Kendall test, even at p<0.05). Linear models fitted through records of mean annual temperatures produced very high temperature increase rates up to 2 K per decade. Only for one pixel (0.1% of all pixels), a significant decreasing temperature trend was determined. Twenty-five percent of the area saw total annual precipitation increase significantly (at p<0.05), whereas decreases in precipitation were found for 5% of pixels. The overall climatic trend over the past 25 years for the Tibetan Plateau was thus a strong warming trend, combined with a moderate trend towards wetter conditions.

### Trends and Climate Responses of Spring Phenology

Few significant trends were detected for the beginning of the growing season and the derived onset of green-up. This finding likely reflects results by Yu et al. [28], who showed that the onset of the growing season advanced until the mid-1990s, before retreating in subsequent years. These opposing trends do not result in a significant trend over the entire study period. According to the PLS analysis, delays in plant spring phases in many pixels were related to warm temperatures during March and February, as well as December of the previous year. These effects may be related to the vernalization requirements of Tibetan grasses, whose fulfillment may be delayed by high temperatures [28]. The strong indications of phenology-advancing effects of warming in spring (April through June) reflect the responsiveness of vegetation to heat after fulfilling winter chilling requirements. In addition to chilling and forcing effects during the winter and spring, warm conditions also appeared to have a delaying effect on BGS dates when they occurred during the previous August and an advancing effect during the previous October. These statistical correlations may be due to temperature effects during dormancy induction, which have been shown to influence chilling requirements in trees [47]. Precipitation effects were less pronounced, but in general high precipitation during most months appeared to advance spring phenology.

Vegetation activity during spring phases was enhanced for the majority of pixels, with weak increases during the green-up phase and slightly greater increases for almost the entire study region during the phase of fast growth. Overall, spring phases were not shifted significantly over the whole study period, but both spring phases showed increases in NDVI.

### Trends and Climate Responses of Summer and Fall Phenology

The timing of plant maturity was advanced in some parts of the Tibetan Plateau. For the end of the growing season and the derived beginning of plant senescence, these advances were very widespread and reached more than 10 days per decade for many pixels. These trends were accompanied by increases in vegetation activity over most of the plateau during the mature phase. This trend was even more pronounced during plant senescence, when NDVI values over much of the plateau increased by more than 0.025 per decade. Primary drivers of these advances were warm conditions in spring and summer (April through August for plant maturity dates; and February, March, April, July and August for the end of the growing season). Only high temperatures in September and October were related to later occurrence of end-of-season phenological stages. High NDVI during maturity and senescence was correlated with low temperatures in May and June, as well as high temperatures during July and September. This may indicate that rapid plant development during early vegetation phases, driven by warm conditions, limits biomass accumulation during later stages. Once plants have reached their mature stage, warm conditions enhance biomass production. Overall, the summer and fall stages of plants have advanced over the past 25 years, accompanied by higher photosynthetic capacity.

### General Discussion

On the Tibetan Plateau, temperature increases over the past 25 years have evidently extended the period, during which temperatures are conducive to plant growth, yet local vegetation seems unable to exploit these additional thermal resources. Instead, Tibetan grasslands have shown little change in the beginning of the growing season, which in recent years appears to have retreated rather than advanced [28]. In combination with advances in summer and fall stages, these trends have led to a significant shortening of the growing season on the Tibetan Plateau [48]. At the same time, photosynthetic capacity, as indicated by the NDVI, has increased significantly during the same time span. Taken together, these trends in vegetation timing and intensity show that vegetation in this region grows faster and more vigorously as temperatures increase. It also reaches the end of its reproductive cycle earlier than in the past, which may be due to the determinate nature of grassland phenology on the Tibetan Plateau. Plants in this winter-cold climate must complete their annual cycles quickly to escape early frosts. Recent temperature increases have brought about earlier fulfillment of their thermal time requirements, after which plants start senescing.

The inability of plants to make full use of available thermal resources hints at a mismatch between current vegetation and climate that is likely due to recent climate change. Current vegetation in most places on the Tibetan Plateau may no longer be the climax vegetation of its environment, and grasses from elsewhere might be better suited to conditions of such places. However, these better adapted plants are not currently available there. Tibetan grasses are largely perennial grasses which predominately propagate vegetatively, so that the speed at which they can extend their habitat through migration is very slow. Under such conditions, it may take many years until a new equilibrium between climate and vegetation is reached. This time lag may increase further if the warming trend of the Tibetan Plateau persists.

For vegetation assemblages that are not well adjusted to the climate that they occur in, projecting future change trajectories is difficult. In particular, using general ecological models for such projections is not necessarily a valid approach. Past studies have shown that it is possible to relate ecological indicators, such as net primary productivity or carbon sequestration, to climatic factors [49], [50], but such relationships may only be valid for ecosystems in their climax state. Yet most ecosystems will take a considerable amount of time to adapt to a new climate regime, and projections about future shifts in climate indicate that many ecosystems may continue lagging behind their climatic setting for many centuries to come. This prospect raises doubts about climate change impact projection methods based on equilibrium states, such as empirical species distribution modeling [51]or approaches based on generalized ecological indicators, such as Dynamic Vegetation Models [52]. Accurately modeling succession processes in ecosystems caused by climate change, in particular the speed, with which various species can adjust to changing environments, is one of the major challenges in climate change impact projection. It will require detailed understanding of species migration, dispersal and climatic responses during all seasons.

### Conclusions

In principle, climatic conditions on the Tibetan Plateau have become more conducive to plant growth over the past 25 years, and the period of thermally favorable conditions has extended. However, plants have not been able to exploit these resources, showing a shorter and more intense growing season rather than a longer period of active growth. Slow species migration rates in the perennial grasslands of the Tibetan Plateau are likely responsible for a mismatch between climate and vegetation assemblage. The current lack of a vegetation-climate equilibrium state makes it difficult to predict climate change impacts on Tibetan grasslands. Our analysis has shown that changes in the timing of phenological events or vegetation activity cannot easily be attributed to individual climate factors, but arise from a combination of different weather conditions during different parts of the growing season. Even weather conditions during the winter months, when plants are dormant, show correlations with the timing of phenological events, and with vegetation activity, which may be related to vernalization requirements. Partial Least Squares regression has proven to be a valuable tool for detecting complex relationships between vegetation and climate and shows promise for analysis of similar situations elsewhere.

## Supporting Information

Figure S1 **Correlations of monthly temperatures (left) and precipitation (right) with the timing of grassland maturity on the Tibetan Plateau, according to Partial Least Squares (PLS) regression.** For each variable, pixels for which the variable-importance-in-the-projection score was<0.8 are shown in gray. Pixels with insufficient data for PLS analysis are shown in white.
(TIF)

Figure S2 **Correlations of monthly temperatures (left) and precipitation (right) with vegetation activity during the period of fast growth (as measured by the NDVI) on the Tibetan Plateau, according to Partial Least Squares (PLS) regression.** For each variable, pixels for which the variable-importance-in-the-projection score was<0.8 are shown in gray. Pixels with insufficient data for PLS analysis are shown in white.
(TIF)

Figure S3 **Correlations of monthly temperatures (left) and precipitation (right) with vegetation activity during plant maturity (as measured by the NDVI) on the Tibetan Plateau, according to Partial Least Squares (PLS) regression.** For each variable, pixels for which the variable-importance-in-the-projection score was<0.8 are shown in gray. Pixels with insufficient data for PLS analysis are shown in white.
(TIF)
