# Supplementary figures and images for: Seasonal Response of Grasslands to Climate Change on the Tibetan Plateau
Source: PLoS One. 2012 Nov 16;7(11):e49230. doi: 10.1371/journal.pone.0049230 (PMC3500274; doi:10.1371/journal.pone.0049230)

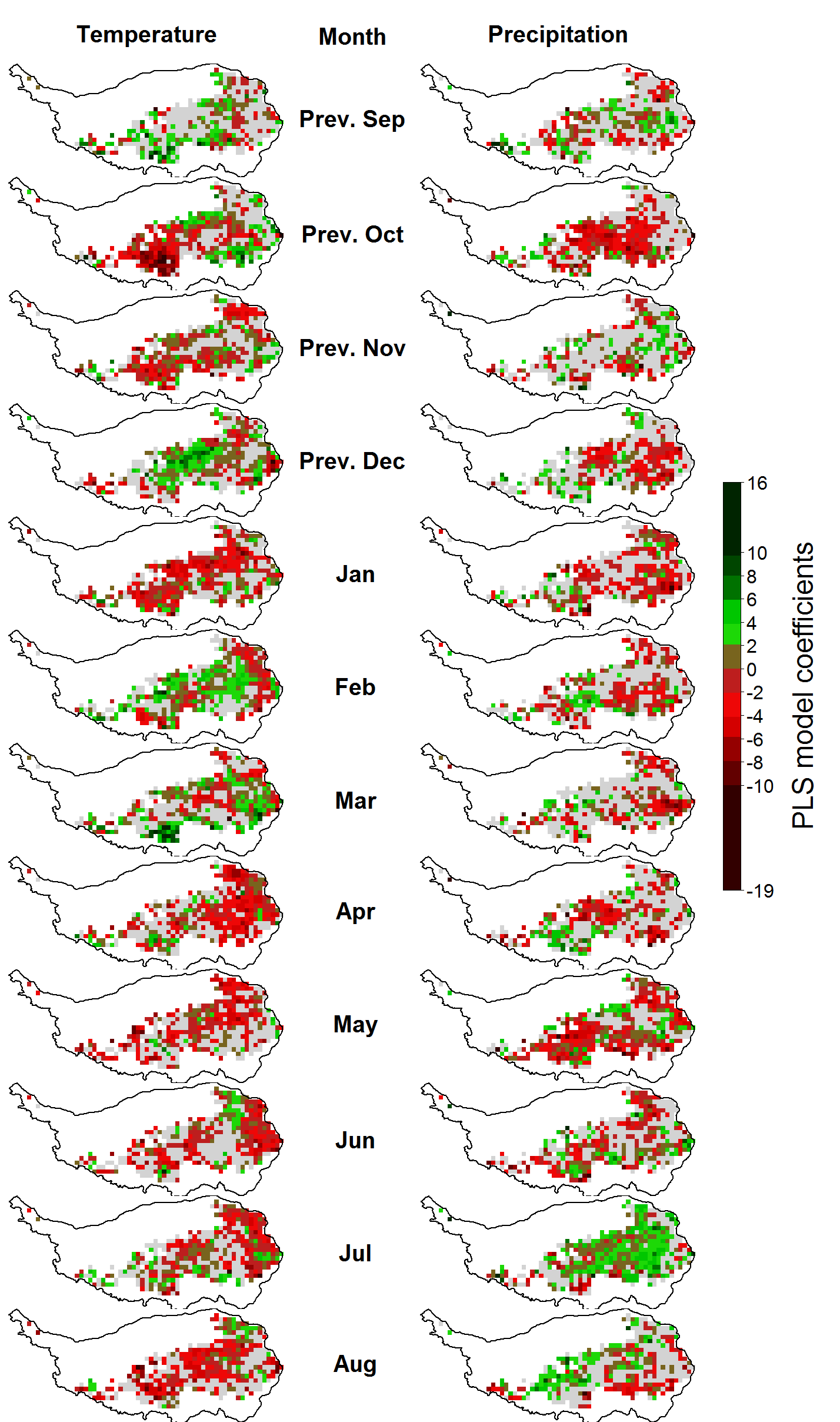

Supplement: Figure S1 — Correlations of monthly temperatures (left) and precipitation (right) with the timing of grassland maturity on the Tibetan Plateau, according to Partial Least Squares (PLS) regression. For each variable, pixels for which the variable-importance-in-the-projection score was<0.8 are shown in gray. Pixels with insufficient data for PLS analysis are shown in white. (TIF) [file pone.0049230.s001.tif]

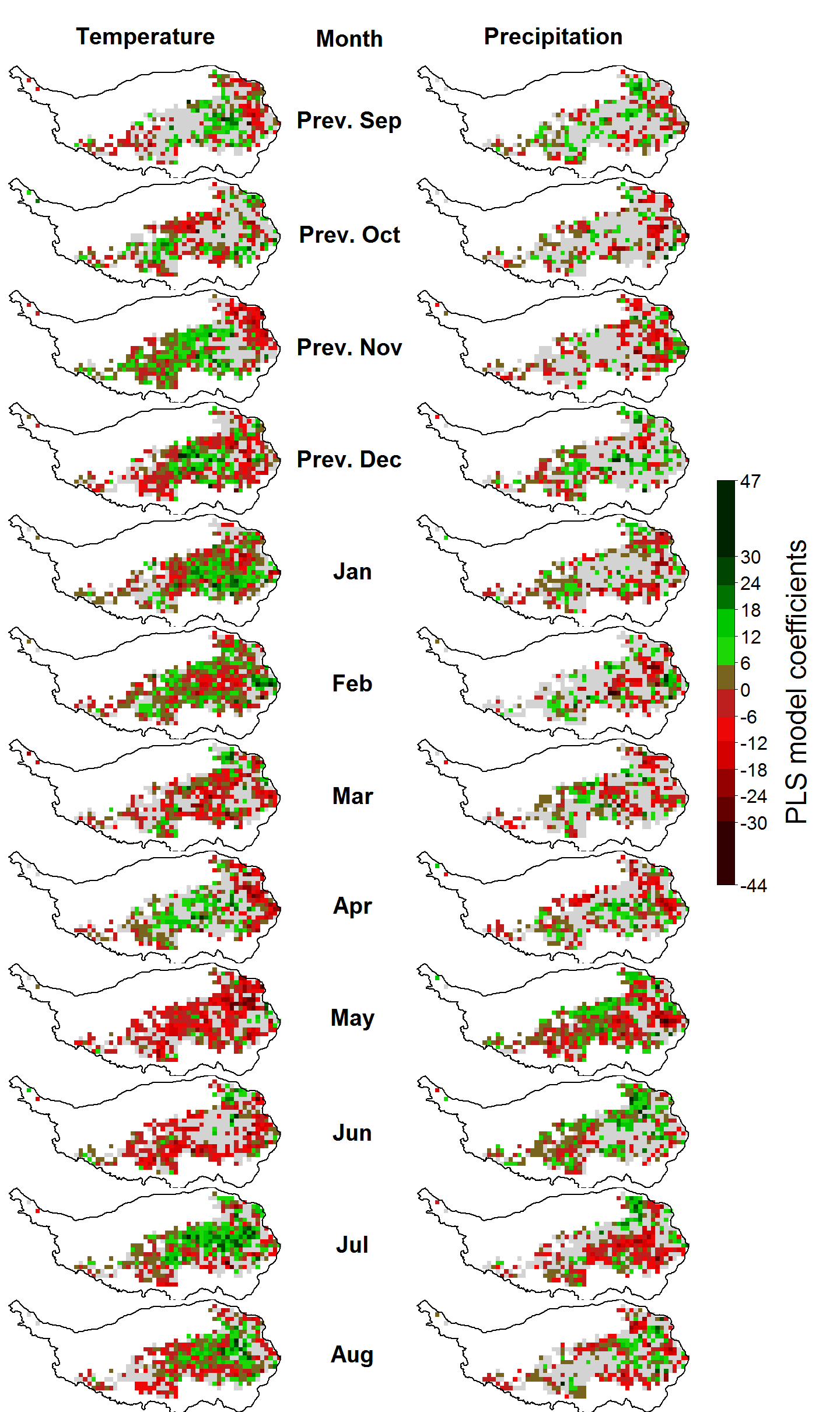

Supplement: Figure S2 — Correlations of monthly temperatures (left) and precipitation (right) with vegetation activity during the period of fast growth (as measured by the NDVI) on the Tibetan Plateau, according to Partial Least Squares (PLS) regression. For each variable, pixels for which the variable-importance-in-the-projection score was<0.8 are shown in gray. Pixels with insufficient data for PLS analysis are shown in white. (TIF) [file pone.0049230.s002.tif]

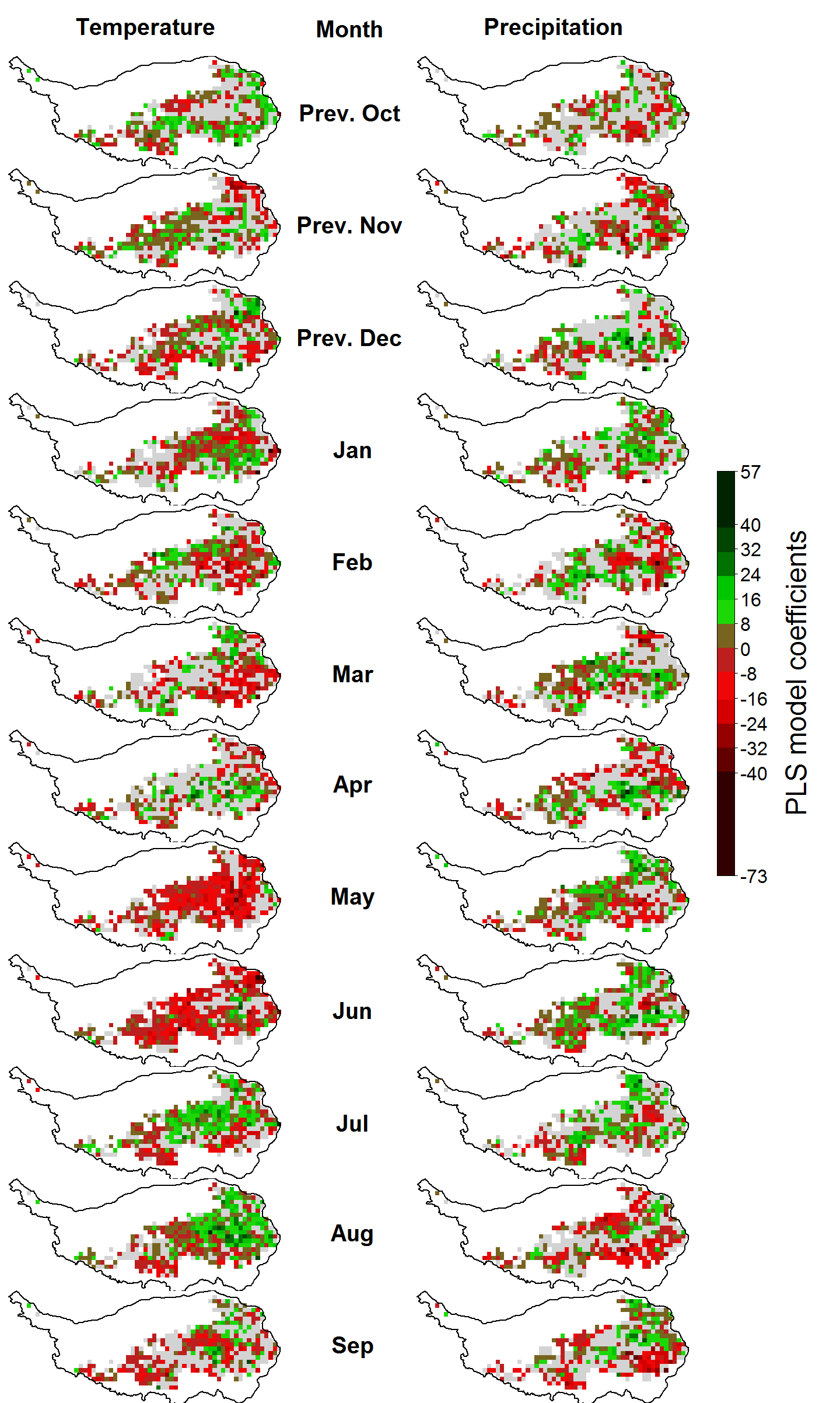

Supplement: Figure S3 — Correlations of monthly temperatures (left) and precipitation (right) with vegetation activity during plant maturity (as measured by the NDVI) on the Tibetan Plateau, according to Partial Least Squares (PLS) regression. For each variable, pixels for which the variable-importance-in-the-projection score was<0.8 are shown in gray. Pixels with insufficient data for PLS analysis are shown in white. (TIF) [file pone.0049230.s003.tif]
